# Case Report: Primary intestinal histiocytic sarcoma in a cat

**DOI:** 10.3389/fvets.2025.1727437

**Published:** 2026-01-08

**Authors:** Andrea Pérez, Jordi Clanxet, Francisco Clemente-Vicario, Jordi Puig, Luis Feo

**Affiliations:** 1 AniCura Ars Veterinaria Hospital Veterinari, Barcelona, Spain; 2 La Merced Veterinary Specialists, Calpe, Spain

**Keywords:** feline oncology, histiocytic sarcoma, immunohistochemistry, intestinal neoplasia, lomustine chemotherapy

## Abstract

Histiocytic sarcoma (HS) is a rare and aggressive tumor in cats, usually presenting in a multicentric or disseminated form, with gastrointestinal involvement mainly reported as part of a metastatic disease. This report describes the first documented case of a primary intestinal HS in an 11-year-old male neutered domestic short-haired cat, presenting with acute gastrointestinal signs. Diagnostic workup included ultrasonography, cytology, histopathology, and immunohistochemistry (IHC), which confirmed a histiocytic origin through Iba-1, CD204 and MHC-II positivity and MUM1, CD117 and Desmin negativity. The patient underwent surgical excision via enterectomy, followed by adjuvant lomustine chemotherapy, achieving complete remission. Despite transient chemotherapy-induced myelosuppression, the cat remains alive and disease-free 2 years after diagnosis. This case highlights the importance of combining imaging, histopathology, and IHC when diagnosing feline intestinal masses.

## 1 Introduction

Histiocytic diseases have been identified in both dogs and cats, encompassing a variety of disorders that differ in clinical presentation, biological behavior, and treatment response. In some cases, distinguishing these neoplasms from granulomatous inflammatory processes or other malignancies such as lymphoma is challenging based only on histopathology (1, 2). However, the use of immunophenotyping has improved diagnostic accuracy by allowing the detection of lineage-specific markers and the classification of histiocytic subtypes (3).

Histiocytes originate from CD34 + precursors and differentiate into macrophages or dendritic cell lineages, including Langerhans cells and interstitial dendritic cells (4). These cells play a crucial role in antigen presentation and are distributed throughout epithelial and connective tissues. In cats, histiocytic diseases originate from either dendritic cells or macrophages and appear in various clinical forms (3). Two feline histiocytic disorders have no canine equivalent: feline progressive histiocytosis (FPH) and pulmonary Langerhans cell histiocytosis (LCH). The FPH is the most common form, is considered a neoplasm of interstitial dendritic cell origin and presents as cutaneous nodules or plaques and is regarded as a low-grade variant of histiocytic sarcoma with an indolent course, predominantly affecting middle-aged cats (5). Conversely, LCH affects older cats (10–15 years) and causes progressive respiratory failure due to diffuse pulmonary infiltration by Langerhans cells (6).

Although HS is well recognized in dogs, it remains rare in cats (2). To date, histiocytic sarcoma in cats has been mainly described through isolated case reports involving various anatomical sites, including the mediastinum with abdominal metastasis, mediastinum with vertebral canal invasion, nasal cavity, intracranial region, bilateral kidneys, oral cavity with or without metastasis, periarticular tissues, as well as the liver and spleen (7–15). The fact that reports are limited to individual cases across different locations makes it difficult to establish a reliable prognosis or determine the best treatment approach. Reported treatments include prednisone and, in some cases, using lomustine as an adjuvant therapy (7, 12–14).

To date, gastrointestinal involvement has generally been reported in cases of disseminated disease rather than as an isolated intestinal condition tumors. Here we describe the first documented case of a primary intestinal histiocytic sarcoma in a cat, confirmed through histopathology and IHC, and successfully managed with enterectomy and adjuvant lomustine chemotherapy.

## 2 Case description

An 11-year-old male neutered domestic short-haired cat was presented to the Internal Medicine Service with an acute onset of projectile vomiting, constipation, hyporexia, and lethargy, which had begun 3 days before presentation. The cat was adopted 5 years ago from a shelter and lived indoors with another healthy cat. There were no previous medical issues, and tests for feline immunodeficiency virus (FIV) antibodies and feline leukemia virus (FeLV) antigen were negative on the SNAP Combo Plus Test (Idexx®).

On physical examination, a non-painful, soft-tissue mass was palpated in the mid-abdomen with no other abnormalities detected. No cutaneous manifestations were observed in the patient. The complete blood count (CBC) revealed a mild, normochromic, normocytic, non-regenerative anemia with a hematocrit of 27.5% (reference range: 30.3–52.3%), while serum biochemical analysis and urinalysis were unremarkable. Abdominal ultrasound demonstrated a mass in the ileocecal valve causing intestinal obstruction, with no other abnormalities in the rest of the abdominal organs. Three-view thoracic radiographs revealed no abnormalities findings.

**Figure 1:**
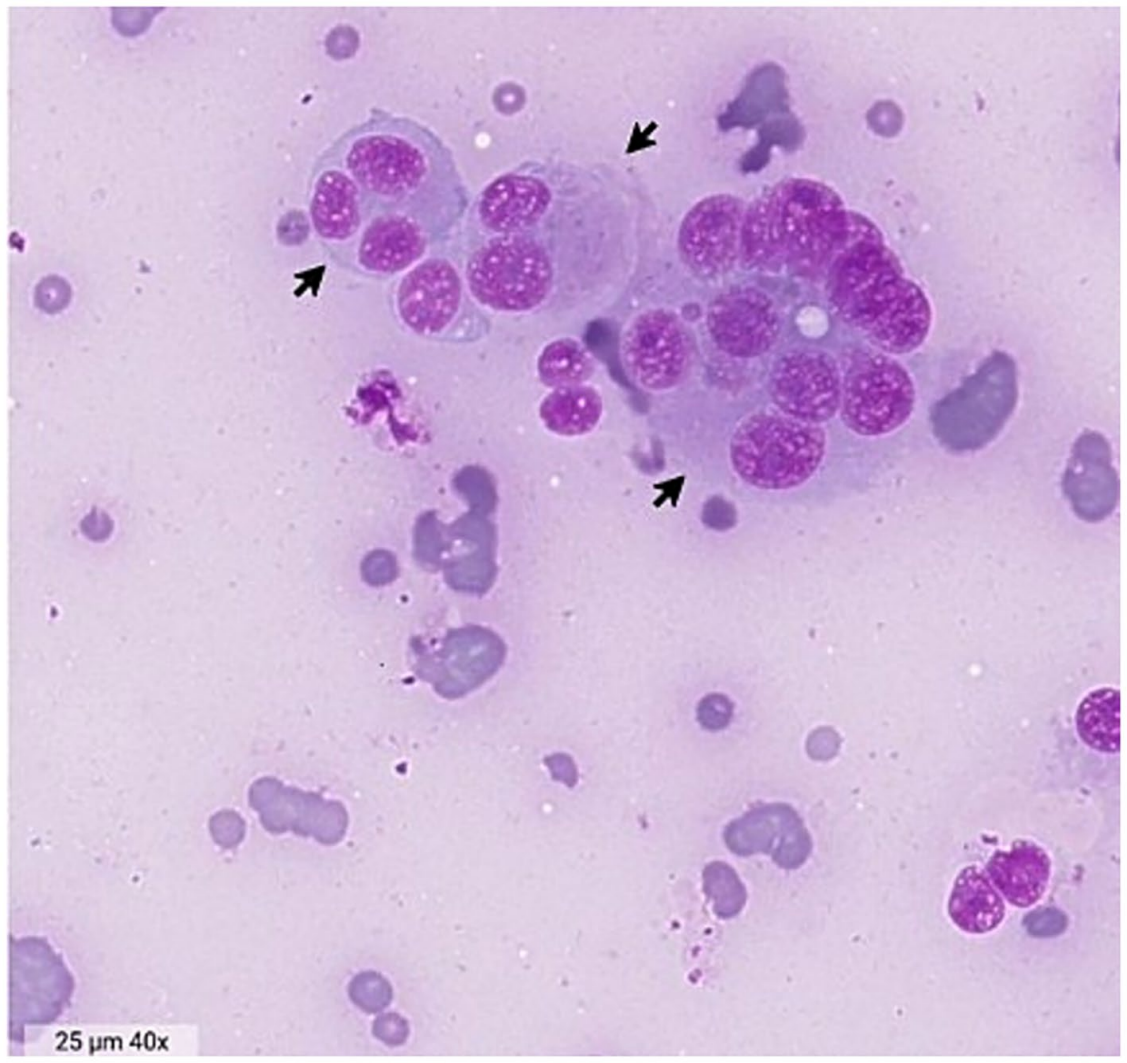
Cytology obtained by fine-needle aspiration of the intestinal mass. Black arrows indicate multinucleated cells. Image from Zoetis VetScan Imagyst® Digital Cytology, 40 X magnification, Diff Quick.

A fine-needle aspiration of the mass was performed, and cytological evaluation, reviewed by a board-certified pathology specialist, revealed numerous neoplastic round to ovoid cells with distinct cell borders on a pale basophilic to moderately hemodiluted background. The cells displayed one to more than 10 round to ovoid, and rarely lobulated, nuclei with occasional small nucleoli. Nuclei were located paracentrally to eccentrically, with moderate to abundant pale basophilic cytoplasm. Low numbers of small lymphocytes, non-degenerate neutrophils, and rare eosinophils were also present (Figure 1). These findings suggested a malignant discrete cell neoplasm, including undifferentiated mast cell tumor, histiocytic sarcoma, and plasma cell neoplasia as differential diagnoses.

**Figure 2:**
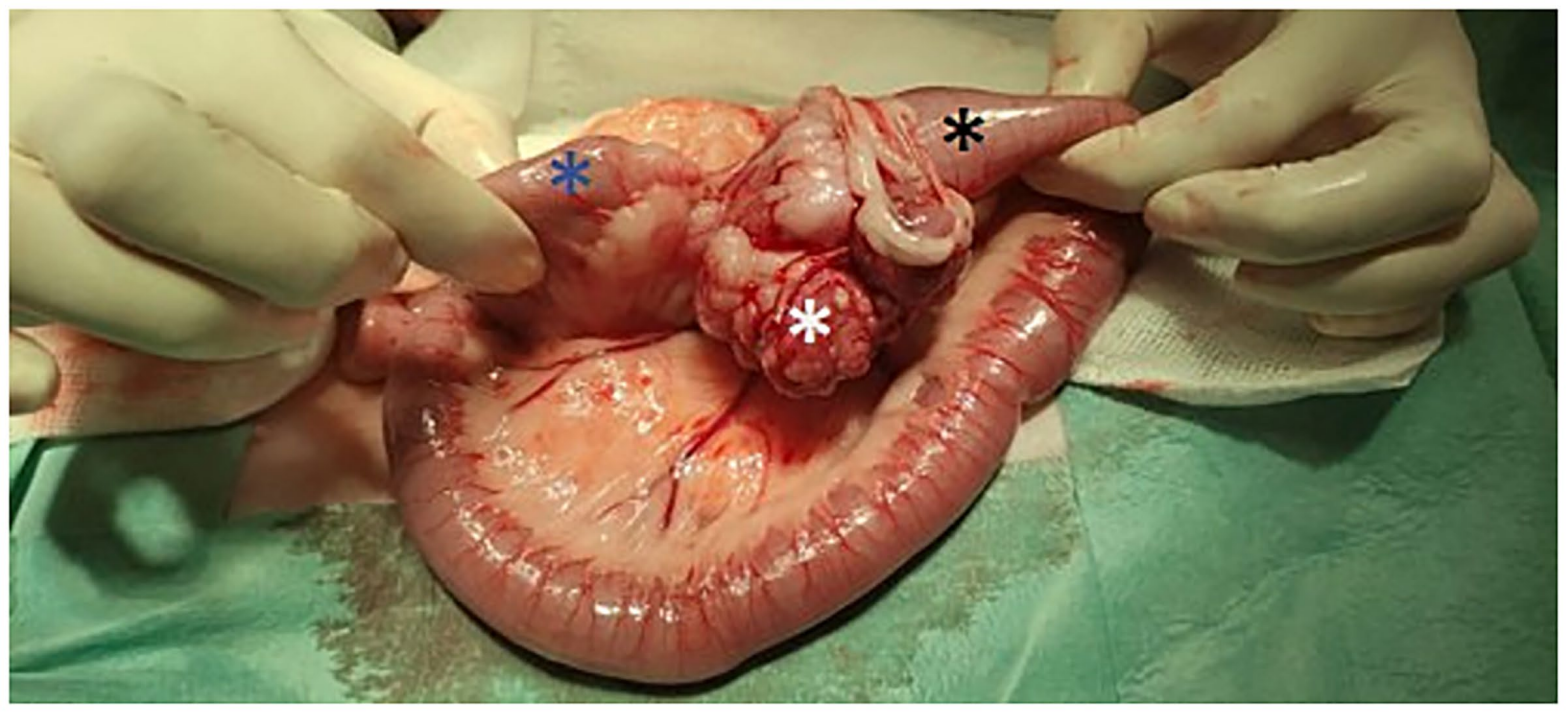
Macroscopic appearance of the intestinal mass at the time of surgery. The black asterisk indicates the ileum, the white asterisk indicates the cecum, and the blue asterisk indicates the colon.

Exploratory laparotomy revealed an approximately 5-cm irregular intestinal mass located just cranial to the ileocecal valve and a slightly enlarged mesenteric lymph node. Enterectomy with resection of the ileocecal valve and lymphadenectomy of the regional lymph node were performed without complications (Figure 2) (16). The cat received amoxicillin-clavulanate (22 mg/kg TID IV) (Normon®), maropitant (1 mg/kg SID IV) (Prevomax® - Dechra, Spain), and methadone (0.2 mg/kg every 4 h IV) (Semfortan® - Dechra, Spain) during hospitalization. The cat was discharged 48 h after surgery with the following oral medications: amoxicillin-clavulanate (22 mg/kg BID PO) (Clavaseptin® - Vetoquinol, Spain), maropitant (2 mg/kg SID PO) (Cerenia® - Zoetis, Spain) and buprenorphine (15 μg/kg TID) (Bupredine® - Dechra, Spain).

**Figure 3:**
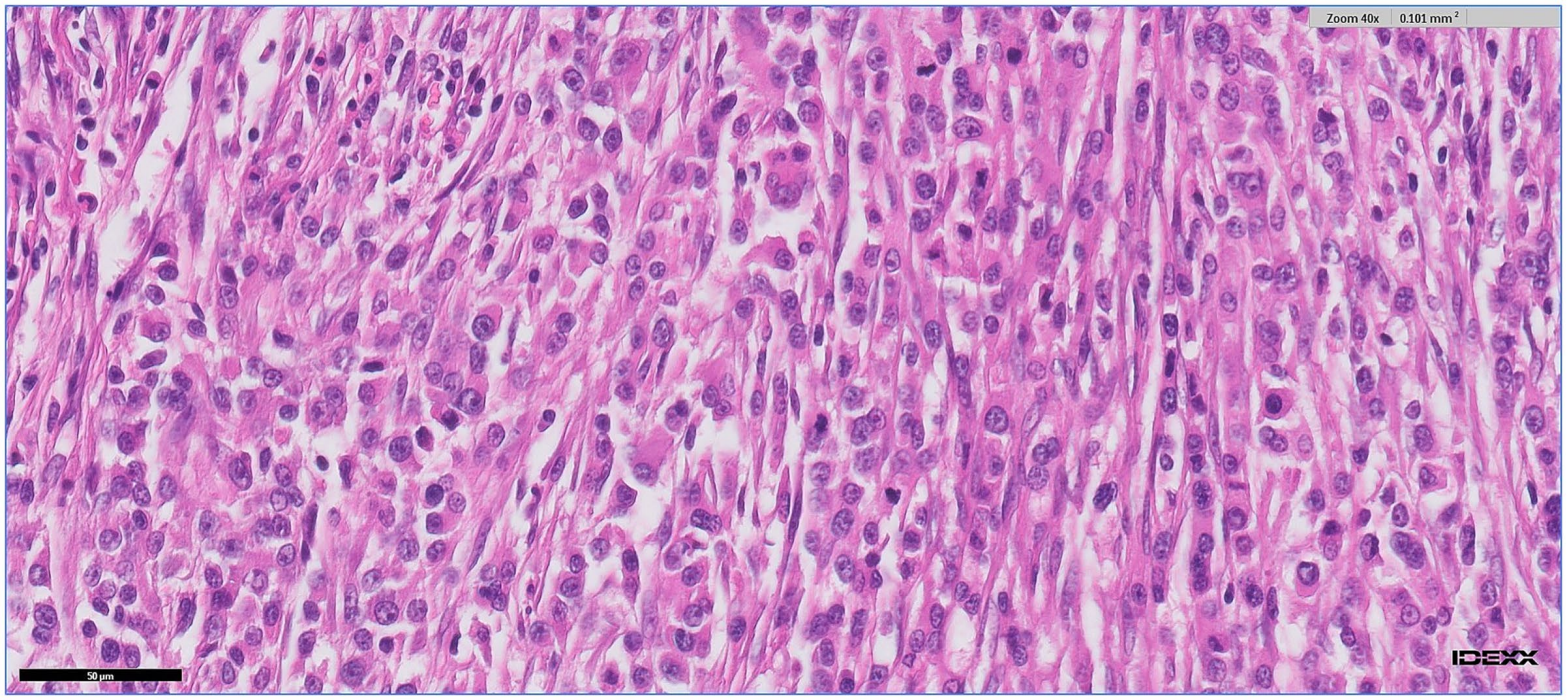
Histopathological section of the intestinal mass. Image from Idexx®, 40 X magnification, Hematoxylin and eosin staining.

Histopathological examination of the mass, reviewed by a board-certified pathology specialist, showed extensive structural distortion, with superficial mucosal erosion replaced by fibrinonecrotic debris and bacterial colonies. A dense, diffuse proliferation of atypical mononuclear cells replaced the mucosa and submucosa and extended into the muscularis. The neoplastic cells had scant eosinophilic cytoplasm, occasional fine granularity, large nuclei with coarse chromatin, and prominent nucleoli. The mitotic count was approximately 46 per 2.37 mm^2^ area, and multinucleated forms were common. Eosinophils, macrophages, necrotic areas, edematous stroma, and septal fibrosis were also present. Partial disruption of the serosa was observed (Figure 3). Histopathological examination of the regional lymph node showed reactive lymphoid hyperplasia, with no evidence of malignant cells metastasis.

**Figure 4:**
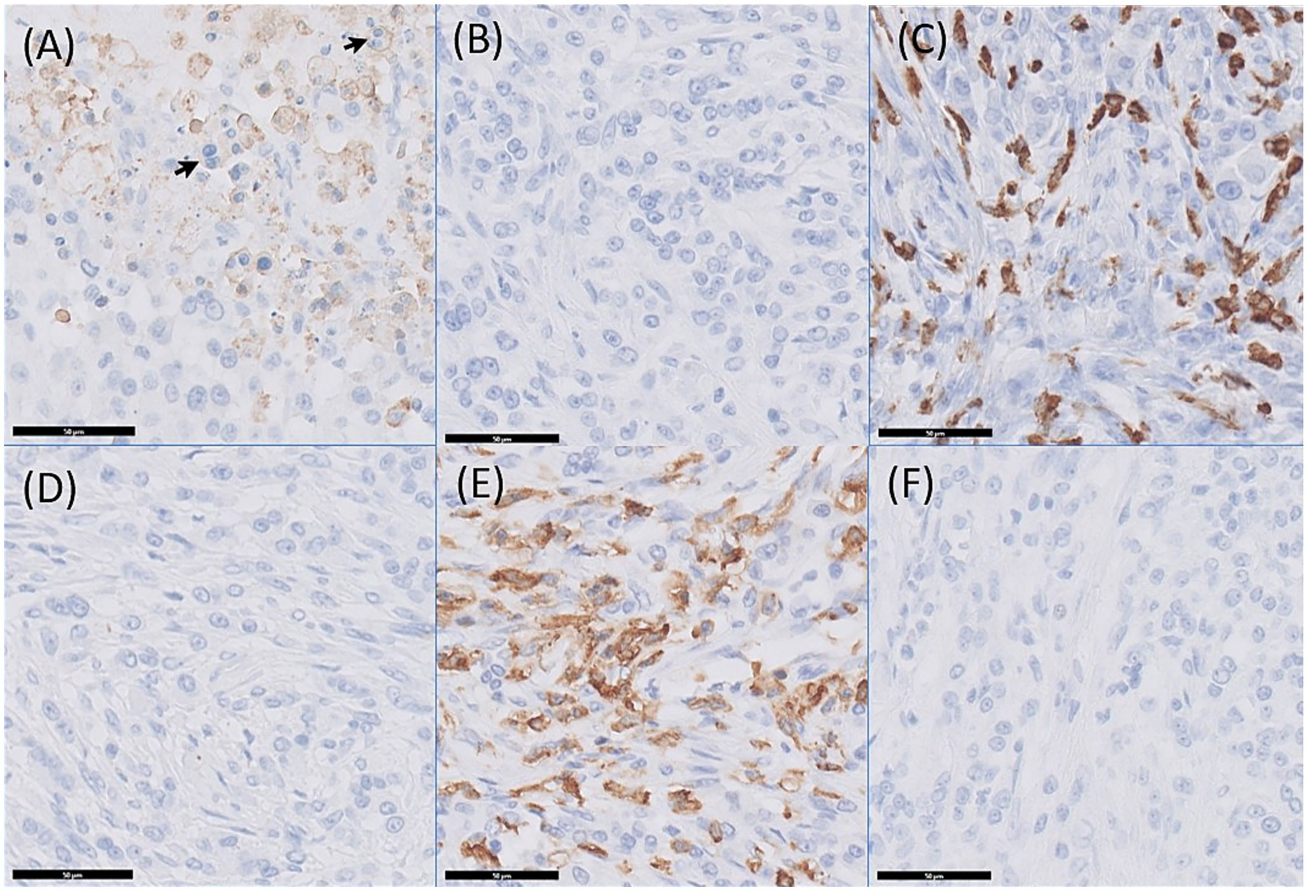
Immunohistochemical staining of the mass: **(A)** Positive cytoplasmic and membranous immunolabeling for Iba-1. Black arrows indicate immunolabeled multinucleate neoplastic cells; **(B)** Negative immunolabeling for CD117; **(C)** Positive cytoplasmic and membranous immunolabeling for CD204; **(D)** Negative immunolabeling for Desmin; **(E)** Positive immunolabeling for MHC-II; **(F)** Negative immunolabeling for MUM-1. Image from Idexx®, 40 X magnification.

Toluidine blue staining was negative for neoplastic mast cells, although scattered mast cells within the inflammatory infiltrate were present. Immunohistochemistry for Iba-1, CD117, MUM1, CD204, anti-Desmin and MHC-II was performed. Neoplastic cells did not show immunolabeling for MUM1, which argues against plasmacytic or lymphoid differentiation. In contrast, immunolabeling for Iba-1 revealed mild, multifocal cytoplasmic and membranous immunolabeling, specifically localized to multinucleated cells and mitotic figures, supporting histiocytic differentiation. The neoplastic cells were negative for Desmin and CD117, excluding mast cell and muscular origin. In contrast, MHC-II showed focal labeling, both as individual cells and as dense aggregates, within a population presumed to correspond to the previously described homogeneous cells. These cells exhibited specific immunolabeling in approximately 65–70% of the lesion. Additionally, CD204 demonstrated cytoplasmic and membranous immunolabeling in neoplastic areas, involving approximately 50–60% of the cells, further confirming histiocytic lineage. Collectively, the observed immunophenotypic profile, in conjunction with the distinctive morphological features, is consistent with a diagnosis of histiocytic sarcoma and allows for the exclusion of other neoplasms, including mast cell tumors, melanomas, and lymphoid malignancies (17, 18) (Figure 4).

One month after surgery, a follow-up blood test was performed, which showed resolution of the anemia (hematocrit 32%). Its resolution following surgical intervention effectively excludes an association with a systemic histiocytic process. The anemia was most likely attributable to the underlying inflammatory state and the potential presence of a subtle, subclinical intraintestinal hemorrhage that was not apparent in the feces. The biochemical profile revealed no significant abnormalities. After reviewing the IHC results, the cat was started on prednisolone (1 mg/kg SID PO) (Prednicortone® - Dechra. Spain) and lomustine (10 mg/cat PO; 33 mg/m^2^) (CeeNu®), with plans for monthly administration. After 4 weeks of treatment, the cat exhibited no clinical signs; however, the CBC showed mild neutropenia with 2.1 × 10^3^/μL (reference range: 2.30–10.9 × 10^3^/μL) and thrombocytopenia with 105 × 10^3^/μL (reference range: 151–600 × 10^3^/μL), consistent with grade 1 immunosuppression according to the Veterinary Comparative Oncology Group (VCOG) toxicity scale (19). Lomustine administration was therefore delayed by 1 week, and the dose was maintained at 10 mg/cat (33 mg/m^2^). Abdominal ultrasound and thoracic radiographs done at this time showed no evidence of recurrence.

Five weeks after the second dose of lomustine, the cat was re-examined and was clinically stable except for mild constipation. Abdominal ultrasonography showed no signs of recurrence, while the CBC revealed thrombocytosis of 730 × 10^3^/μL (reference range: 151–600 × 10^3^/μL). The third dose of lomustine was administered (10 mg/cat; 33 mg/m^2^), and the dose of prednisone was reduced to 0.5 mg/kg.

Three weeks after administering lomustine, the cat showed petechiae on physical examination of the inner pinnae and abdomen. The CBC revealed significant neutropenia (0.61 × 10^3^/μL) and thrombocytopenia (4 × 10^3^/μL), while serum biochemistry, abdominal ultrasound, and thoracic radiographs were unremarkable. Myelosuppression caused by lomustine was suspected and aligned with grade 3 immunosuppression according to the Veterinary Comparative Oncology Group (VCOG) toxicity scale (19). The cat was started on amoxicillin-clavulanate 22 mg/kg every 12 h, with no change in the prednisolone dose.

At recheck 10 days after starting antibiotics, clinical signs had resolved and no petechiae were observed. The CBC showed mild non-regenerative anemia with a hematocrit of 27.5% (reference range: 30.3–52.3%), persistent neutropenia at 0.17 × 10^3^/μL, and a normal platelet count at 264 × 10^3^/μL. Antibiotics were continued for an additional 7 days. On subsequent evaluation, the CBC again revealed mild non-regenerative anemia (28.2%), mild neutropenia (1.93 × 10^3^/μL), and a normal platelet count (563 × 10^3^/μL). At this point, the owner elected to discontinue further chemotherapy. Prednisone was gradually tapered until complete discontinuation.

Restaging was conducted at 8 and 24 months after diagnosis, including comprehensive laboratory tests, hematology, biochemistry, abdominal ultrasonography, and thoracic radiographs. On both occasions, no evidence of relapse was found, and the patient remains in remission.

## 3 Discussion

This report represents the first documented case of a solitary histiocytic sarcoma originating within the feline intestine. The tumor’s confinement to the intestinal tract, without evidence of systemic dissemination, highlights an uncommon manifestation of this neoplasm and provides new insights into its potential biological behavior in cats. Such an atypical presentation expands the current understanding of histiocytic sarcoma in this species and underscores the importance of considering it among the differential diagnoses for feline intestinal masses.

According to Kehl et al. (20), approximately 13% of all feline tumors are exclusively located within the intestine, emphasizing the relative infrequency of intestinal neoplasia compared with tumors in other anatomical sites. Among these, lymphoma is recognized as the most prevalent intestinal neoplasm in cats, followed by adenocarcinomas and, less frequently, sarcomas and other tumor subtypes (20). Previous reports of histiocytic sarcoma in cats have largely consisted of isolated cases affecting a variety of anatomical locations (7–15), and descriptions of its occurrence within the gastrointestinal tract remain exceedingly uncommon.

Diagnostic accuracy in histiocytic sarcomas can be challenging, as the sensitivity and specificity of some ancillary tests are not definitive. Misclassification with other round-cell tumors remains a concern, although in this case, the morphological and immunohistochemical features strongly supported the diagnosis. One of the possible differential diagnoses to be excluded, given the presence of eosinophils within the tissue, was granulomatous inflammation. However, several histopathological and immunohistochemical features supported the diagnosis of histiocytic sarcoma instead. The neoplastic population was composed of pleomorphic, large round to polygonal cells with marked anisocytosis and anisokaryosis, exhibiting high mitotic activity and evidence of cellular atypia, which are not consistent with a granulomatous process. Immunohistochemistry in particular played a crucial role, providing high-quality evidence that distinguished histiocytic sarcoma from morphologically similar neoplasms such as lymphomas or poorly differentiated sarcomas.

Across the available studies evaluating lomustine administration in cats, hematologic toxicity has been consistently identified as the primary dose-limiting adverse effect (21, 22). In early phase I and clinical evaluations, neutropenia emerged as the most common and clinically relevant hematologic toxicity, while thrombocytopenia occurred less frequently but could still reach clinically significant grades. In our case, the occurrence of neutropenia and thrombocytopenia requiring supportive treatment led the owners to discontinue chemotherapy due to concerns about potential adverse effects. It is possible that a protocol involving less frequent administration or a pre-adjusted, individualized dose, rather than a standard fixed dose of 10 mg, might have helped to prevent the development of these adverse events.

Remarkably, the clinical outcome in this case has been positive. Surgical removal, initially combined with lomustine chemotherapy, achieved excellent disease control, even though chemotherapy had to be stopped early due to adverse effects. The use of lomustine was based on previous studies in dogs, where it showed effectiveness against histiocytic sarcoma, with response rates reported between 29 and 46% (23). So far, all published feline cases have described histiocytic sarcoma as an aggressive disease with a consistently poor prognosis, usually leading to death or euthanasia. Our case, therefore, represents the first documented remission in a cat, suggesting that, at least in cases of intestinal localization, surgical management alone can provide significant therapeutic benefit. The role of chemotherapy in this case remains unclear, as it was started after the surgery; however, the potential benefit of lomustine in non-surgical cases is acknowledged (23). Nonetheless, the possibility of late relapse cannot be ruled out, emphasizing the importance of long-term monitoring and follow-up.

## 4 Conclusion

In summary, this case emphasizes the diagnostic value of IHC in veterinary oncology and highlights the need to consider histiocytic sarcoma in the differential diagnosis of feline intestinal masses, in addition to lymphoma and carcinoma. Combining imaging techniques, histopathological examination, and IHC was crucial to reaching a definitive diagnosis. Although the rarity of this disease has hindered the development of an optimal treatment protocol in veterinary literature, this report shows that localized intestinal histiocytic sarcoma in cats may have a better prognosis than previously thought, indicating potential for individualized treatment strategies. However, more cases need to be studied to draw definitive conclusions.

## Data Availability

The original contributions presented in the study are included in the article/supplementary material, further inquiries can be directed to the corresponding authors.
